# COVID-19: a retrospective cohort study with focus on the over-80s and hospital-onset disease

**DOI:** 10.1186/s12916-020-01665-z

**Published:** 2020-06-25

**Authors:** Simon E. Brill, Hannah C. Jarvis, Ezgi Ozcan, Thomas L. P. Burns, Rabia A. Warraich, Lisa J. Amani, Amina Jaffer, Stephanie Paget, Anand Sivaramakrishnan, Dean D. Creer

**Affiliations:** 1 Department of Respiratory Medicine, Barnet Hospital, Royal Free London NHS Foundation Trust, Wellhouse Lane, London, EN5 3DJ UK; 2 Department of Microbiology, Barnet Hospital, Royal Free London NHS Foundation Trust, Wellhouse Lane, London, EN53DJ UK

**Keywords:** COVID-19, Coronavirus, Elderly, Hospital acquired, Ethnicity

## Abstract

**Background:**

Data from the UK COVID-19 outbreak are emerging, and there are ongoing concerns about a disproportionate effect on ethnic minorities. There is very limited information on COVID-19 in the over-80s, and the rates of hospital-onset infections are unknown.

**Methods:**

This was a retrospective cohort study from electronic case records of the first 450 patients admitted to our hospital with PCR-confirmed COVID-19, 77% of the total inpatient caseload to date. Demographic, clinical and biochemical data were extracted. The primary endpoint was death during the index hospital admission. The characteristics of all patients, those over 80 years of age and those with hospital-onset COVID-19 were examined.

**Results:**

The median (IQR) age was 72 (56, 83), with 150 (33%) over 80 years old and 60% male. Presenting clinical and biochemical features were consistent with those reported elsewhere. The ethnic breakdown of patients admitted was similar to that of our underlying local population. Inpatient mortality was high at 38%.

Patients over 80 presented earlier in their disease course and were significantly less likely to present with the typical features of cough, breathlessness and fever. Cardiac co-morbidity and markers of cardiac dysfunction were more common, but not those of bacterial infection. Mortality was significantly higher in this group (60% vs 28%, *p* < 0.001). Thirty-one (7%) patients acquired COVID-19 having continuously been in hospital for a median of 20 (14, 36) days. The peak of hospital-onset infections occurred at the same time as the overall peak of admitted infections. Despite being older and more frail than those with community-onset infection, their outcomes were no worse.

**Conclusions:**

Inpatient mortality was high, especially among the over-80s, who are more likely to present atypically. The ethnic composition of our caseload was similar to the underlying population. While a significant number of patients acquired COVID-19 while already in hospital, their outcomes were no worse.

## Background

In December 2019, a febrile respiratory tract illness was reported in a cluster of patients in Wuhan City (Hubei Province, China) [1] which we now recognise as the novel pathogenic strain of coronavirus (SARS-coronavirus-2 [SARS-CoV-2]) [2]. The World Health Organization subsequently declared the coronavirus disease 2019 (COVID-19) a public health emergency of international concern [3]. The infection has spread rapidly across the globe with nearly 6.5 million infections reported worldwide and over 380,000 deaths by 25 April [4].

The first laboratory-confirmed case of COVID-19 in the UK was reported on January 30, 2020, [5] with a subsequent rapid rise in the number of cases nationally. As of 4 June 2020, 281,661 patients have tested positive for the disease and a total of 39,904 have died [6]. The London peak occurred some 2 to 3 weeks ahead of much of the rest of the UK.

The first COVID-19 cases in Barnet were reported on 5 March 2020. Barnet is the largest London borough by population with a 2017 population estimate of 406,600 inhabitants [7]. Barnet Hospital, a busy suburban hospital with 440 beds, confirmed its first PCR-positive COVID-19 patient on 9 March 2020. Since this, initial case numbers have risen rapidly with the number of laboratory confirmed COVID-19 inpatients admitted at 587 by 25 April. The peak daily number of positive tests on inpatients was 48, on 2 April, and the number of confirmed COVID-19 inpatients peaked at 274 on 6 April.

Since the early case reports, there have been numerous publications from China [8], the USA [9] and elsewhere [10] describing presenting features and outcomes of the disease and, more recently, from the UK [11]. However, few prior publications have examined the presentation of COVID-19 in the over-80s, and none have reported rates of hospital-onset infections. There are also significant concerns in the UK about an apparent excess in COVID-19-related mortality among ethnic minorities [12].

We hypothesised that COVID-19 would disproportionately affect older patients and that these patients would be more likely to present atypically and also that there would be a significant proportion of patients who acquired the condition while already hospitalised.

In order to investigate this, we analysed our first 450 laboratory-confirmed cases of COVID-19. This comprises 77% of our cases during the first UK peak as of 25 April. We examined the demographics, ethnicity, clinical and biochemical features, presentations in older adults and hospital-onset infections. We also aimed to provide a detailed and complete picture of the disease as it might present to a busy suburban general hospital. This will provide useful information as services in the UK are remodelled in the run-up to lifting of restrictions and a possible second peak of infections.

## Methods

Inpatients returning consecutive positive polymerase chain reaction (PCR) tests for SARS-CoV2 on nasopharyngeal swabs during their hospital admission were included for analysis. Data were collected retrospectively from the electronic patient record. Patients with a clinical diagnosis of COVID-19 without PCR confirmation were not included. Details of the PCR testing methodology are included in the supplementary data appendix.

Standardised data were collected on demographic features, ethnicity and the presence of co-morbidities (prior diagnosis of cardiac disease [any], hypertension, diabetes, respiratory disease [any] and immunosuppression). In those patients over 65 years of age, the Clinical Frailty Score (CFS) [13] was recorded where available. The presence of care needs prior to admission, including carers at home and institutional care, was recorded.

Community-onset infection was defined as a positive test within 14 days of hospital admission and hospital-onset infection if the patient had continuously been an inpatient for the 14 days prior to the positive PCR test. Data were recorded at the point of presentation, defined as the day of hospital admission (community-onset infections) or documentation of first symptom presentation in the medical notes (hospital-onset infections). Clinical data included symptom duration and presenting symptoms and signs. A fever was defined as a temperature > 37.8 °C.

Biochemical data included serum lymphocyte and neutrophil counts, C-reactive protein (CRP), procalcitonin, cardiac troponin T, lactate, D-dimer and glucose. The presence of acute kidney injury was defined according to 2012 Kidney Disease: Improving Global Outcomes guidance [14]. These tests were analysed by the hospital clinical laboratory; further details including normal limits and detection thresholds are included in the supplementary appendix (Additional file 1: Table S1). Values outside the detection thresholds were entered at the threshold.

The primary outcome assessed was death vs discharge from hospital alive at the end of the hospital episode, where the patient had reached this point. Some ventilated patients were transferred to other centres, and outcome data was unavailable at the time of analysis. Early outcomes at day 5 following presentation were also captured and defined as discharged, remaining inpatient, intubated inpatient or dead. Other outcomes included length of stay and whether antibiotics were given.

Most variables were not expected to be normally distributed and were reported as median (interquartile range [IQR]). Non-parametric tests were used throughout. Continuous between-group variables were analysed using the Wilcoxon signed-rank test. The Bonferroni correction for multiple analyses was used for comparisons within tables. Categorical variables were analysed using the chi-squared test. Analysis was performed using R Statistics version 3.6.3.

## Results

### Demographics, clinical characteristics and outcomes

Four hundred seventy-six positive swabs were identified; 26 were excluded as they were either too young (less than 16 years old) or not admitted as inpatients to the hospital. Four hundred fifty inpatients who underwent consecutive PCR tests confirming COVID-19 between 10 March 2020 and 8 April 2020 were analysed. This represents 77% of the PCR-positive caseload admitted to our hospital to date.

Table 1 displays the demographic and clinical characteristics of the patients, subdivided by outcome. The median (IQR) age was 72 (56, 83) years, and in keeping with the elderly population local to our hospital, two thirds were over the age of 60 and one third over 80. There was a male predominance. Patients who died were significantly older (median (IQR) age 80 (72, 88) vs 61 (49, 79), *p* < 0.001) and more likely to be receiving care in the community (69 (40%) vs 45 (19%), *p* < 0.001), with a trend towards greater frailty by CFS. Of the 45 (10%) patients who were admitted from care homes, 35 (78%) died during their admission and only 10 (22%) survived to discharge. These patients had a high median age of 84 (78, 91) and median clinical frailty score of 7 (6, 7).

**Table 1:** Demographics and baseline characteristics by outcome of hospital admission

	All patients (***n*** = 450)	By outcome of hospital stay, where available (***n*** = 410)		***p*** value for comparison
		Discharged (***n*** = 237)	Died (***n*** = 173)	
**Demographics**				
Age in years, median (IQR)	72 (56, 83)	61 (49, 79)	80 (72, 88)	< 0.001*
Age breakdown, *n* (%)				
< 40	32 (7)	29 (12)	2 (1)	–
40–59	105 (23)	81 (34)	10 (6)	–
60–79	163 (36)	70 (30)	71 (41)	–
> 80	150 (33)	57 (24)	90 (52)	–
Male gender, *n* (%)	272 (60)	134 (57)	111 (64)	0.146
BMI, median (IQR)	26 (24, 30)	27 (24, 30)	25 (23, 31.5)	0.214
Receiving care prior to admission, *n* (%)	118 (26)	45 (19)	69 (40)	< 0.001*
Care home, *n* (%)	45 (38)	10 (4)	35 (20)	–
Own home + carers, *n* (%)	49 (42)	21 (9)	26 (15)	–
Other care, *n* (%)	24 (20)	14 (6)	8 (5)	–
CFS if > 65 years, median (IQR)	5 (3, 6)	4 (3, 5.5)	5 (3, 6)	0.014
Ever smoker, *n* (% of available data)	76 (31)	50 (35)	20 (26)	0.228
COVID-19 acquired in hospital, *n* (%)	31 (7)	20 (8)	7 (4)	0.117
Ethnicity, *n* (%)				0.072**
Asian	51 (11)	31 (13)	13 (8)	–
Black	33 (7)	19 (8)	11 (6)	–
White	265 (59)	127 (54)	118 (68)	–
Other	77 (17)	42 (18)	26 (15)	–
Unavailable	24 (5)	18 (8)	5 (3)	–
PCR test returning positive, *n* (%)				0.59**
First	410 (91)	214 (90)	158 (91)	
Second	34 (8)	19 (8)	14 (8)	
Third	6 (1)	4 (6)	1 (1)	
**Pre-existing comorbidities,*n*(%)**				
Hypertension	195 (43)	87 (37)	90 (52)	0.0029
Cardiac condition	141 (31)	55 (23)	78 (45)	< 0.001*
Diabetes	134 (30)	68 (29)	53 (31)	0.589
Respiratory condition	85 (19)	47 (20)	34 (20)	1.00
Immunosuppression	42 (9)	27 (11)	13 (8)	0.251
**Disease characteristics**				
Symptoms at presentation, *n* (%)				
Cough	317 (70)	173 (73)	113 (65)	0.228
Breathlessness	282 (63)	142 (60)	113 (65)	0.204
Diarrhoea	70 (16)	38 (16)	28 (16)	1.00
Symptom duration at presentation in days, median (IQR)	5 (2, 8)	5 (2, 9)	4 (2, 7)	0.109
Signs at presentation				
Respiratory rate	24 (20, 30)	23 (19, 28)	26 (22, 33)	< 0.001*
SaO2, median (IQR)	94 (90, 96)	95 (92, 96)	92 (88, 95)	< 0.001*
(*n* [%] measured on supplemental O2)	235 (52)	106 (45)	106 (61)	
Heart rate, median (IQR)	90 (80, 103.5)	90 (80, 103.5)	90 (78.25, 100)	0.970
Systolic BP < 100	38 (8)	16 (7)	19 (11)	0.180
Median temperature	37.9 (37.2, 38.4)	38 (37.3, 38.5)	37.8 (37.2, 38.1)	0.004
Fever > 37.8	262 (58)	143 (60)	88 (51)	0.081
Abnormal chest radiograph, *n* (%)	370 (82)	181 (76)	153 (88)	0.015
Antibiotics given, *n* (%)	332 (74)	165 (70)	136 (79)	0.049
Length of stay in days, median (IQR)	7 (3, 11)	7 (4, 12)	6 (3, 10)	0.127
Length of stay in days, median (IQR)	7 (3, 11)	7 (4, 12)	6 (3, 10)	0.127
Status at day 5, *n* (%)				
Discharged	79 (18)	79 (33)	0 (0)	–
Non-intubated inpatient	243 (54)	146 (62)	77 (45)	–
Intubated	56 (12)	10 (4)	26 (15)	–
Died	70 (16)	0 (0)	70 (40)	–

Comparisons between those who died and those who were discharged used the Kruskal-Wallis test or the chi-squared test as appropriate. The Bonferroni method was used to correct for multiple comparisons, and therefore, a stringent *p* value cutoff of 0.05/25 = 0.002 was used to assess significance (indicated by *)

**Differences were assessed by *χ*^2^ test to examine differences in overall composition between groups

Thirty-one patients (7%) acquired COVID-19 while already admitted to hospital.

Fifty-nine percent overall were white, similar to the 59.7% of the local population who were classified as ethnically white according to 2020 projections from 2011 census data [7]. There was a trend towards a difference in ethnicity, with a higher proportion of white ethnicity among those who died (*χ*^2^*p* = 0.07), but this reflects documented age-related differences in our local population with a higher proportion of our older residents being white [15].

Hypertension was present in 195 (43%) of patients admitted, with cardiac disease in 141 (31%), diabetes in 134 (30%), respiratory conditions in only 85 (19%) and immunosuppression in 42 (9%). Pre-existing cardiac disease (78 (45%) vs 55 (23%, (*χ*^2^*p* = 0.005)) and hypertension (87 (37%) vs 90 (52%, (χ^2^*p* = 0.003)), but not diabetes, respiratory conditions or immunosuppression were significantly more prevalent in those patients who died than those who were discharged. All types of cardiac disease, respiratory disease and immunosuppression were included; breakdowns of prevalence of the different disease types are listed in the supplementary appendix (Additional file 1: Tables S2, S3 and S4 respectively).

Cough and breathlessness were reported at presentation in 317 (70%) and 282 (63%) respectively. Diarrhoea was reported by 70 (16%) patients. The median duration of symptoms was 5 (2, 8) days. At presentation, those who died had a higher respiratory rate (26 (22, 33) vs 23 (19, 28), *p* < 0.001) and lower oxygen saturations (92 (88, 95) vs 95 (92, 96), *p* < 0.001) than those discharged. Presenting temperature, heart rate and the presence of hypotension were no different between these groups although there was a trend towards lower median temperature in those who died. The majority (74%) of patients received antibiotic therapy for a median (IQR) duration of 5 (3, 7) days.

**Fig. 1:**
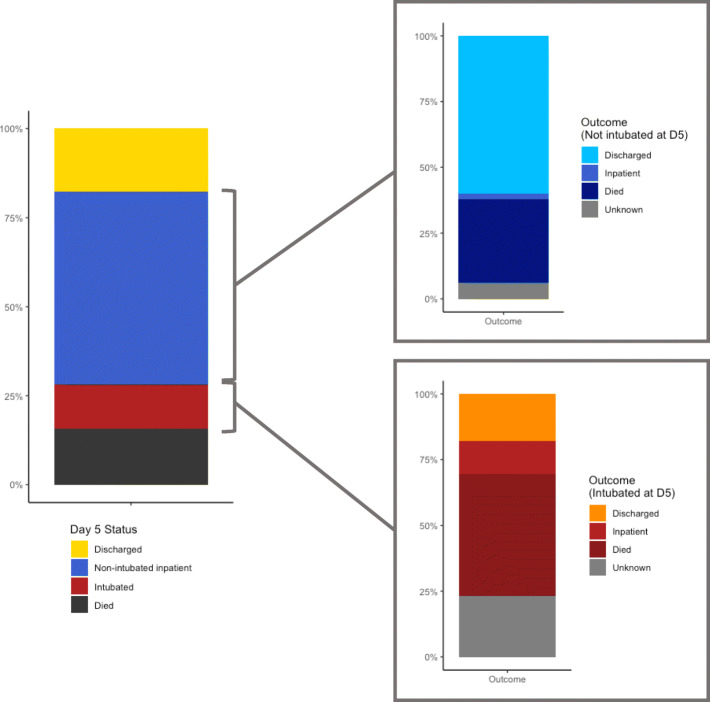
Outcomes for first hospital admission by status at day 5

At day 5 following admission, 79 (18%) of patients had been discharged, 243 (54%) were not intubated but remained in hospital, 56 (12%) were intubated and receiving mechanical ventilation and 70 (16%) had died. Of those intubated at day 5, 26 (46%) had died, 10 (18%) had gone home, 13% remained in hospital and 13 (23%) had unknown outcomes, usually following transfer to another centre (Fig. 1). The median length of stay was 5 (2, 8) days.

At the time of data analysis, 173 (38%) patients had died, 237 (53%) had been discharged, 12 (3%) remained in hospital and 28 (6%) had unknown outcomes.

### Biochemical disease characteristics by outcome

Table 2 summarises the biochemical presenting features overall and by outcome of hospital stay.

**Table 2:** Laboratory studies at presentation with COVID-19, subdivided by the outcome of the hospital stay where available (*n* = 410)

	All patients (***n*** = 450)	By outcome of hospital stay, where available (***n*** = 410)		*p* value for comparison
		Discharged (***n*** = 237)	Died (***n*** = 173)	
Lymphocyte count	0.84 (0.58, 1.23)	0.91 (0.67, 1.28)	0.78 (0.535, 1.14)	0.009
Neutrophil count	5.72 (3.84, 8.61)	5.32 (3.48, 7.82)	6.6 (4.178, 9.750)	0.001*
Neutrophil: lymphocyte ratio	221.5 (112.8, 333.2)	217 (117, 323)	239 (106.5, 343.5)	0.676
CRP	99 (46, 176.5)	68 (31, 140)	131 (74, 199)	< 0.001*
CRP > 100, *n* (%)	221 (49)	83 (35)	108 (62)	< 0.001*
Procalcitonin	0.26 (0.13, 0.73)	0.20 (0.11, 0.44)	0.37 (0.17, 1.35)	< 0.001*
Troponin	23 (9, 50)	12 (6, 33)	42 (20, 71.5)	< 0.001*
Lactate	1.3 (0.9, 1.8)	1.2 (0.9, 1.6)	1.5 (1.1, 2.25)	< 0.001*
D-dimer	1294 (616.5, 2429.8)	1186 (535, 2340)	1577 (814, 2548)	0.014
Glucose	6.6 (5.8, 8.3)	6.5 (5.575, 8.0)	6.9 (5.9, 8.625)	0.017
Acute kidney injury, *n* (%)	85 (19)	24 (10)	54 (31)	< 0.001*

Comparisons between those who died and those who were discharged used the Kruskal-Wallis test or the chi-squared test as appropriate. The Bonferroni method was used to correct for multiple comparisons, and therefore, a stringent *p* value cutoff of 0.05/12 = 0.0042 was used to assess significance (indicated by *)

Inflammatory markers that differed significantly between those that died and those that were discharged were neutrophil count (6.6 (4.178, 9.750) vs 5.32 (3.48, 7.82), *p* = 0.001), CRP (131 (74, 199) vs 68 (31, 140), *p* < 0.001), procalcitonin (0.37 (0.17, 1.35) vs 0.20 (0.11, 0.44), *p* < 0.001), cardiac troponin (1422 (506, 4473) vs 12 (6, 33), *p* < 0.001) and lactate (1.5 (1.1, 2.25) vs 1.2 (0.9, 1.6), *p* < 0.001). Acute kidney injury (24 (10%) vs 54 (31%), (*χ*^2^*p* < 0.001)) was more prevalent in those who died.

### COVID-19 in the over-80s

One hundred fifty (33%) patients were aged 80 years or over, and the characteristics of COVID-19 in these patients were examined. The median (IQR) age in this group was 86 (83, 91), and the oldest was 101. Seventy (53%) were male.

When compared with those patients under 80 years, these patients were more frail (median (IQR) CFS 5 (4,6 vs 3 (2, 5), *p* < 0.001)), more likely to have been receiving care prior to admission (76 (51%) vs 42 (14%), *χ*^2^*p* < 0.001), and had lower body mass index (BMI) (median (IQR) 24 (21, 40) vs 28 (25, 32), *p* < 0.001). Ethnicity breakdown was significantly different in the over-80s (χ^2^*p* < 0.001), with the proportions of White patients higher than in the overall caseload (72%), likely related to the age-related ethnic composition of our older local population.

Prior diagnosis of a cardiac condition was significantly more common in those > 80 years (79 (53%) vs 62 (21%), *χ*^2^*p* < 0.001), as was hypertension (76 (51%) vs 119 (40%), *χ*^2^*p* = 0.02) but diabetes, respiratory conditions and immunosuppression were not.

Patients over 80 were significantly less likely to present with the typical syndrome of breathlessness (72 (48%) vs 210 (70%), χ^2^*p* < 0.001), cough (87 (58%) vs 230 (77%), *χ*^2^*p* < 0.001) or fever (75 (50%) vs 186 (62%), χ^2^*p* = 0.02). Median (IQR) respiratory rate was lower at presentation (23 (19, 28) vs 26 (20, 32) *p* = 0.001), as was heart rate (84.5 (77.25, 96.75) vs 95 (80, 106), *p* < 0.001). The median (IQR) symptom duration prior to presentation was significantly lower in the older group (4 (1, 6.25) vs 7 (2, 9) days, *p* < 0.001).

Biomarker profiles were compared between the older and younger groups for those biomarkers listed in Table 2. Median (IQR) troponin (56 (35.5, 94.5) vs 14 (7, 32), *p* < 0.001), D-dimer (1966 (1136, 2768) vs 1120 (538, 2058), p < 0.001), and lactate (1.45 (1.1, 2.1) vs 1.3 (0.9, 1.7)) were significantly higher in the over-80s while the lymphocyte count was lower (0.76 (0.53, 1.14) vs 0.89 (0.62 vs 1.28), *p* = 0.007). Notably there were no differences in CRP, procalcitonin, neutrophil count or glucose between age groups.

Mortality was significantly higher in the older age group (90 (60%) vs 83 (28%), *χ*^2^*p* < 0.001). The over-80s that died were more frail (median (IQR) CFS 6 (5, 7) vs 5 (4, 6), *p* = 0.002). Median (IQR) respiratory rate (24 (20, 30) vs 21 (18, 25.25), *p* < 0.001) and heart rate (88 (78, 99) vs 83 (74, 88), *p* = 0.03) were significantly higher in those that died. Median (IQR) CRP was significantly higher in those that died than those that survived (125 (73.25, 199.75) vs 67.50 (25.75, 119), *p* < 0.001)); other biomarkers were not significantly different.

### Hospital-onset infections

**Fig. 2:**
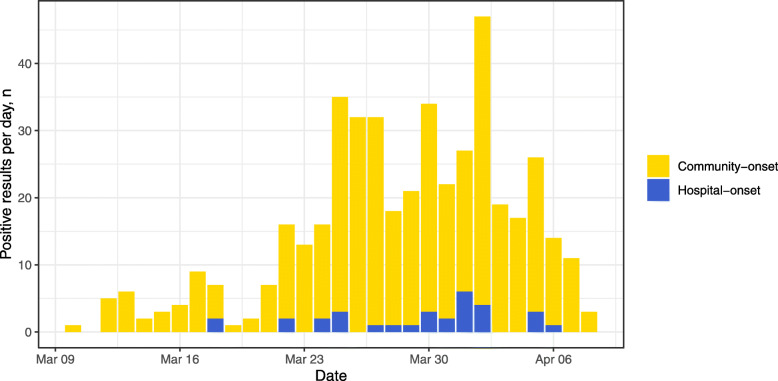
Timeline of cases of community-onset and hospital-onset COVID-19 in patients admitted to hospital

Thirty-one (7%) of infections were hospital-onset. The median (IQR) duration of hospital stay prior to COVID-19 testing was 20 (14, 36) days. The first hospital-onset infection was recorded 8 days after the first positive test on an inpatient; the peak of hospital-onset infections occurred approximately 3 weeks later and mirrored that of the community-onset infections (Fig. 2).

These patients were older (median (IQR) age 80 (72.5, 88.5) vs 71 (55.5, 83), *p* = 0.002), slightly more frail (median (IQR) CFS 5 (4, 6) vs 5 (3, 6), *p* = 0.047) and more likely to have required care prior to admission (17 (55%) vs 101 (24%), *χ*^2^*p* < 0.001). Median (IQR) symptom duration was much shorter than in community-onset infections (1 (1, 2) vs 5 (2, 8) days, *p* < 0.001) likely reflecting the enhanced monitoring of these hospital inpatients. Median CRP (38.5 (12.25, 72.50) vs 104 (50, 127), *p* < 0.001) was significantly lower in the hospital-onset group.

Seven (23%) patients with hospital-onset infections died compared to 166 (40%) of those with community-onset infections (*χ*^2^*p* = 0.09) suggesting that despite their vulnerability their overall outcomes were no worse. Ten (32%) patients with hospital-onset infections were asymptomatic at the time of their swab, which was performed based on low oxygen saturations or pyrexia

## Discussion

We report here the characteristics of nearly 80% of the patients with confirmed COVID-19 presenting to our hospital during the early part of the UK COVID-19 outbreak. We specifically examined the presentation of disease in the over-80s and those who acquired COVID-19 in hospital.

In-hospital mortality per hospital admission was high at 38%, in line with recently released data from the ISARIC collaboration [11]. This reflects the severity of disease in those hospitalised with COVID-19 as well as the underlying age of our population. However, even in the under-80s, mortality was 28%. The UK experience therefore differs dramatically from the initial reports from China [16], with a reported in-hospital mortality of 1.4%. It is also higher than the 21% reported in the USA by Richardson and colleagues [9], although that population was younger and their follow-up duration shorter meaning that fewer patients may have reached this endpoint by the time of analysis. It seems that, at least in the early stages of the epidemic in Wuhan, all patients with COVID-19 were hospitalised regardless of disease severity.

Hospital practice in the UK has been to only admit those patients medically requiring hospitalisation, and this is therefore a much sicker cohort overall. There was also a hugely increased overall healthcare burden on the hospital during the period of the pandemic peak. A comparison of our admissions during a sample 14-day period (14 March–7 April) during 2020 and 2019 showed a 23% increase in hospital admissions, 63% of which were with PCR-confirmed COVID-19 in 2020. The death rate from these admissions was 43% in 2020 vs 7% in 2019, and the intubation rate was 17% vs 1% (unpublished data). Even though we were unable to assess the direct mechanism of death in these patients, the presence of COVID-19 clearly caused an excess in admissions, mortality and intensive care admissions compared to the previous year.

Presenting characteristics were similar to those recently reported from the UK [11], although our patients were older and more frail than series from elsewhere [16]. Those patients that died during their hospital admission were older, more likely to have been receiving care before admission, and had more cardiac disease than those that did not, again in line with data from ISARIC [11]. Interestingly, the prevalence of diabetes did not differ between these groups, in contrast to other reports [16]. Those that died were more clinically unwell at presentation than those who survived, and biochemically had significantly higher levels of inflammation and markers of cardiac and renal dysfunction, in line with previous reports [16, 17]. While previous studies have noted the neutrophil lymphocyte ratio to be predictive of poor outcome [18], we found that it did not differ by mortality.

There has been much concern in the UK about disproportionately high numbers of people from Black and minority ethnic (BAME) groups being admitted and dying from COVID-19 [12]. We examined this in our dataset. The ethnic profile of the admitted patients (59% White, 41% BAME) was almost identical to that of our underlying population, which in 2020 was projected to be 59.7% White and 40.3% BAME [7]. The percentage of White patients was higher in those who died and was 72% in those over 80 years old, again consistent with the higher proportion of White people in those who are older in the borough [15]. We therefore did not find evidence that the patients admitted or dying with COVID-19 differed from our local population although this study was not powered or designed to look at this specifically. Barnet is a relatively affluent borough compared to others in London, and it is also possible that this may have reduced any component of mortality related to socioeconomic status.

Previous studies of COVID-19 in the older population are small and have used differing variations for ‘elderly’ ranging from 60 to 65 years [19, 20]; even these have included only small numbers of patients that would be classically considered ‘elderly’ in the UK, usually over 80 years of age. Mortality is linked to increasing age, and in line with non-COVID disease in the elderly, there has been suggestion [21] but little evidence that these patients are more likely to present atypically. Our findings confirm this; patients over 80 were significantly less likely to present with the typical COVID-19 features of cough, breathlessness and fever. They also presented earlier in their disease course suggesting lower physiological reserve. Lymphocyte count was lower in the over-80s, possibly reflecting age-related immune dysfunction or more severe disease.

Age is independently linked to mortality [11] and this is reflected in our findings here. Although troponin differed significantly between age groups, consistent with the higher incidence of cardiac disease in the over-80s, it did not differ significantly by outcome in the elderly population. This may suggest that although cardiac dysfunction is present in the older population it is not the cause of death per se, although the smaller numbers here may mean that a statistically significant association may have been missed. CRP was associated with poor outcome in this age group, but neither this nor procalcitonin differed between the age categories, suggesting that bacterial infection may not be a greater driver of disease in older patients.

It is also notable that a high proportion of the patients admitted from local care homes (78%) did not survive to discharge. These patients were among the most frail in the cohort. Local care homes were asked at the start of the outbreak to avoid hospitalising their patients wherever possible, and these are therefore likely to have been the most unwell of all the care home patients who contracted COVID-19, as well as the overall numbers being lower. The data in this study do not allow us to comment on the risk of contracting COVID-19 in care homes. These differences in the presentation and outcomes in older adults are of utmost importance given the significantly higher mortality seen in this population and the emerging picture of how COVID-19 has affected care homes in the UK. Further research should elucidate the mechanisms by which age-related biological variance impacts on the pathogenic response in COVID-19 and will be vital to enable effective therapeutic interventions.

Previous series reporting the incidence of nosocomial COVID-19 estimate a higher proportion of nosocomial infection (44%) than we found in our patients [22]. Data have been of poor quality, however, and information from outside Hubei is lacking. For our analysis we used a stringent definition, including only those patients with continuous inpatient admission for the whole of the 14-day incubation period prior to symptoms, and the true incidence is almost certainly higher. Likewise, a proportion of the community-onset patients may have been misclassified. We felt it important, however, to be as robust as possible when reporting this important issue.

These patients had already had prolonged admissions for unrelated reasons and correspondingly were an older and more frail group than the population as a whole. Despite this, CRP was lower and their outcomes were no worse, reflecting the fact that they effectively acquired COVID-19 incidentally while in hospital rather than presenting due to severe infection. The peak incidence of hospital-onset infections mirrored the overall peak of community infections, and the mode of transmission remains unclear; this may include relatives visiting before they were excluded and asymptomatic infected healthcare workers. It may also have included transmission from other patients either directly or via healthcare workers. Urgent further research into this area will be crucial to enabling robust infection control policy in the ongoing management of this and future pandemics.

There are several limitations to this study. We did not collect data on some presenting features that are important, notably atypical symptoms at presentation and more detail on pre-existing comorbidities and medications. We also did not formally assess the causality of the deaths that were associated with COVID-19, although our clinical impression is that the majority of the deaths were directly linked. Furthermore, this was an observational study, and therefore, data collection was not standardised. Owing to this, and the fact that we only introduced a clinical care bundle specifying laboratory tests 2 to 3 weeks into the outbreak, there is a proportion of missing data in some of the biochemical variables. This will have affected our ability to detect more subtle signals, although does not diminish the significance of those we have reported. We also did not have follow-up data on those patients discharged and were therefore unable to assess subsequent deaths or readmissions. Despite these limitations, the size of this cohort has allowed us to present a reasonably complete picture of COVID-19 as it presented to our UK hospital.

## Conclusions

We have described the patients admitting to our suburban UK hospital with COVID-19. Inpatient mortality was high, particularly among the over-80s. The presenting features overall were consistent with those reported elsewhere, but the over-80s were more likely to present with atypical symptoms. The ethnic composition of patients appeared to be similar to that of the underlying population. A significant number of patients acquired COVID-19 in hospital, the precise mode of transmission for which remains unknown.

## Supplementary information

**Additional file 1 : Table S1**. Normal ranges and limits of detection for the laboratory markers analysed in this study. **Table S2**. Prevalence of different cardiac conditions in those recorded as having history of cardiac disease. **Table S3**. Prevalence of different respiratory conditions in those recorded as having history of respiratory disease. **Table S4**. Prevalence of different immunosuppressive conditions and treatments in those recorded as being immunosuppressed. **Table S5**. missing data by variable.

## Data Availability

The data analysed during this study are available from the corresponding author on reasonable request.

## Abbreviations

BAME: Black and minority ethnic
BMI: Body mass index
CFS: Clinical Frailty Score
CRP: C-reactive protein
IQR: Interquartile range
PCR: Polymerase chain reaction
