# Supplementary material for: COVID-19: a retrospective cohort study with focus on the over-80s and hospital-onset disease
Source: BMC Med. 2020 Jun 25;18:194. doi: 10.1186/s12916-020-01665-z (PMC7315690; doi:10.1186/s12916-020-01665-z)
Supplement: Supplementary file 1 — Additional file 1 : Table S1. Normal ranges and limits of detection for the laboratory markers analysed in this study. Table S2. Prevalence of different cardiac conditions in those recorded as having history of cardiac disease. Table S3. Prevalence of different respiratory conditions in those recorded as having history of respiratory disease. Table S4. Prevalence of different immunosuppressive conditions and treatments in those recorded as being immunosuppressed. Table S5. missing data by variable. [file 12916_2020_1665_MOESM1_ESM.docx]

**Supplementary data appendix**

**To accompany manuscript entitled ‘COVID-19: a retrospective cohort study with focus on the over-80s and hospital-onset disease’**

**Details of PCR testing for SARS-CoV2**

Initial testing was done centrally at the Public Health England (PHE) Laboratory in Colindale, London, prior to in-house assays being set up in mid-March. Three different RT-PCR (Reverse transcriptase polymerase chain reaction) assays were then used at our laboratory. The choice of the assay depended on staffing, platform and reagent availability.

The three assays used were:

- Abbott m2000 system
- In-house assay performed on the Hologic Panther Fusion platform
- In-house assay performed on the ThermoFisher Quant Studio 5 platform

In-house primers were developed to target the SARS CoV2 N (nucleoprotein) gene. The Abbott commercially available kit also targets the SARS CoV2 N gene. The in-house primers available from Public Health England (PHE) targeted the RdRp (RNA dependent RNA polymerase) gene. Our in-house assays were developed by Health Services Laboratory in collaboration with PHE, and validation reports demonstrated increased sensitivity compared with the assay available at PHE Colindale. For this reason, positive results in our lab were not forwarded to Colindale for confirmation.

A second method of testing directly from swabs was validated by HSL, removing the need for RNA extraction and therefore useful when supplies of extraction reagents were problematic. This carries a slightly decreased sensitivity and is utilised only on samples tested on the ThermoFisher Quant Studio 5 platform.

Verification across the three platforms demonstrated 100% concordance. Validation of samples tested directly from swabs, using the current assays as gold standard, gave a diagnostic sensitivity of 98% and a diagnostic specificity of 100%.

Table S1: Normal ranges and limits of detection for the laboratory markers analysed in this study.

| Biochemical marker | Normal Range |
| --- | --- |
| Lymphocyte count  (x 10⁹/L) | 1.0 – 4·0 |
| Neutrophil count  (x 10⁹/L) | 1.7 – 7·5 |
| CRP  (mg/L) | 0 - 5 |
| Procalcitonin  (µg/L) | 0 - 0·5 |
| Troponin  (ng/L) | <5 |
| Lactate  (mmol/L) | 0·4 - 0·8 |
| D-dimer  (ng/L) | 0 - 500 |
| Glucose  (mmol/L) | 3·5 - 11·1 |

Normal ranges for biochemical markers. Relevant lower limits of detection were noted for procalcitonin (0·1 µg/L) and troponin (3 ng/L). The upper limit of detection for D-dimer was 80,000 ng/L.

Table S2: Prevalence of different cardiac conditions in those recorded as having history of cardiac disease

| Comorbidity | Number of patients with condition (%)* |
| --- | --- |
| AF | 59 (42) |
| Ischaemic Heart Disease** | 49 (35) |
| Stroke/TIA | 30 (21) |
| Heart Failure | 20 (14) |
| Pacemaker | 20 (14) |
| Valve Disease*** | 11 (8) |
| Other**** | 11 (8) |

*52 patients had two or more cardiac co-morbidities

**Ischaemic heart disease includes those documented as having ischaemic heart disease, coronary artery disease, angina, history of myocardial infarction, history of revascularization.

***Valve disease includes those with aortic valve and mitral valve disease

****Includes history of PE, sick sinus syndrome, dilated cardiomyopathy, childhood atrial septal defect repair.

AF=Atrial fibrillation, TIA=transient ischaemic attack

Table S3: Prevalence of different respiratory conditions in those recorded as having history of respiratory disease

| Comorbidity | Number of patients with condition (%)* |
| --- | --- |
| Asthma | 41 (48) |
| COPD | 25 (29) |
| OSA | 9 (11) |
| Lung Cancer | 6 (7) |
| Bronchiectasis | 5 (6) |
| ILD | 3 (4) |
| Other** | 6 (7) |

*12 patients had two or more respiratory co-morbidities

**Includes pulmonary proteinosis, pulmonary sarcoidosis, myasthenia gravis requiring ventilation support, lung granulomas of unknown cause, benign pleural plaques.

COPD=Chronic Obstructive Pulmonary Disease, OSA=Obstructive Sleep Apnea, ILD=Interstitial lung disease

Table S4: Prevalence of different immunosuppressive conditions and treatments in those recorded as being immunosuppressed

| Comorbidity | Number of patients with condition (%)* |
| --- | --- |
| Medications** | 12 (29) |
| Pregnant | 10 (24) |
| Haematological Malignancy*** | 7 (17) |
| Chemotherapy/Immunotherapy | 5 (12) |
| Other**** | 10 (24) |

*Two patients had two immunosuppressive conditions

**Medications included Prednisolone, Mycophenolate Mofetil, Azathioprine

***Multiple Myeloma and Chronic Lymphocytic Leukemia

****Includes renal transplant, splenectomy, unexplained recurrent chest infections, HIV, alcohol excess, non haematological active cancer

Table S5: missing data by variable.

| Study variable | Number missing | Percent missing |
| --- | --- | --- |
| Admission Date | 0 | 0 |
| PCR result Date | 0 | 0 |
| Hospital-onset y/n | 2 | 0 |
| Positive swab number | 0 | 0 |
| Age | 0 | 0 |
| Gender | 0 | 0 |
| Diabetes | 3 | 1 |
| Hypertension | 3 | 1 |
| immunosuppression | 3 | 1 |
| Respiratory Condition y/n | 4 | 1 |
| Clinical Frailty Score (if >65) | 204 | 45 |
| Weight | 181 | 40 |
| Height | 263 | 58 |
| BMI | 265 | 59 |
| Ethnicity | 24 | 5 |
| Smoking History | 203 | 45 |
| Cardiac Disease | 4 | 1 |
| Temperature on admission | 2 | 0 |
| Lymphocyte count | 3 | 1 |
| Neutrophil count | 3 | 1 |
| Procalcitonin | 171 | 38 |
| C-reactive protein | 3 | 1 |
| Troponin | 181 | 40 |
| D-dimer | 228 | 51 |
| lactate | 79 | 18 |
| glucose | 42 | 9 |
| Acute Kidney Injury | 4 | 1 |
| Symptom duration | 16 | 4 |
| Cough | 6 | 1 |
| Breathlessness | 5 | 1 |
| Diarrhoea | 7 | 2 |
| Oxygen saturations | 3 | 1 |
| Respiratory rate | 3 | 1 |
| Heart rate | 147 | 33 |
| Hypotension | 2 | 0 |
| Antibiotic therapy | 6 | 1 |
| Antibiotic duration | 139 | 31 |
| Radiographic abnormalities | 18 | 4 |
| Day 5 outcome | 2 | 0 |
| Length of stay | 43 | 10 |
| Admitted from place of care | 7 | 2 |
| Hospital onset | 0 | 0 |
| Outcome | 0 | 0 |
